# Epigenetic Alteration of the Cancer-Related Gene TGFBI in B Cells Infected with Epstein–Barr Virus and Exposed to Aflatoxin B1: Potential Role in Burkitt Lymphoma Development

**DOI:** 10.3390/cancers14051284

**Published:** 2022-03-02

**Authors:** Francesca Manara, Antonin Jay, Grace Akinyi Odongo, Fabrice Mure, Mohamed Ali Maroui, Audrey Diederichs, Cecilia Sirand, Cyrille Cuenin, Massimo Granai, Lucia Mundo, Hector Hernandez-Vargas, Stefano Lazzi, Rita Khoueiry, Henri Gruffat, Zdenko Herceg, Rosita Accardi

**Affiliations:** 1 International Agency for Research on Cancer, World Health Organization, 69000 Lyon, France; manaraf@students.iarc.fr (F.M.); antoninjay@hotmail.fr (A.J.); odongog@iarc.fr (G.A.O.); audrey.diederichs@gmail.com (A.D.); sirandc@iarc.fr (C.S.); cueninc@iarc.fr (C.C.); khoueiryr@iarc.fr (R.K.); 2 CIRI, Centre International de Recherche en Infectiologie, RNA Expression in Viruses and Eukaryotes Group, Universite Claude Bernard Lyon I, INSERM U1111, CNRS UMR5308, ENS Lyon, 69007 Lyon, France; fabrice.mure@ens-lyon.fr (F.M.); m_maroui@yahoo.com (M.A.M.); 3 Department of Medical Biotechnology, Section of Pathology, University of Siena, 53100 Siena, Italy; gr.massimo@live.it (M.G.); lazzi2@unisi.it (S.L.); 4 Health Research Institute, University of Limerick, V94 T9PX Limerick, Ireland; lucia.mundo.ml@gmail.com; 5 Lyon Cancer Research Center (CRCL), INSERM U1052, Centre Léon Bérard, 69000 Lyon, France; hector.hernandez-vargas@lyon.unicancer.fr

**Keywords:** Burkitt lymphoma, EBV, AFB1, DNA methylation

## Abstract

**Simple Summary:**

Endemic Burkitt lymphoma is an aggressive pediatric cancer whose etiological factors include Epstein–Barr virus (EBV) infection and malaria or environmental carcinogen exposures, such as aflatoxin B1 (AFB1). As evidence suggests B cell methylome remodeling to be a transformation mechanism shared by both EBV and AFB1, the identification of a common molecular signature compromising cell fate could reveal an essential driver of lymphomagenesis and provide a relevant target for novel therapies. We, therefore, explored the genome-wide DNA methylation profiles associated with both endemic Burkitt lymphoma and AFB1 exposure and identified a shared signature affecting the expression of a putative tumor suppressor, TGFBI, whose reduced expression has already been investigated in several cancers, but whose implication in lymphoma has not been evidenced so far. Further research clarifying the functional consequence of TGFBI suppression on B cell fate and the impact on tumor microenvironment reshaping is warranted.

**Abstract:**

Burkitt lymphoma (BL) is a malignant B cell neoplasm that accounts for almost half of pediatric cancers in sub-Saharan African countries. Although the BL endemic prevalence is attributable to the combination of Epstein–Barr virus (EBV) infection with malaria and environmental carcinogens exposure, such as the food contaminant aflatoxin B1 (AFB1), the molecular determinants underlying the pathogenesis are not fully understood. Consistent with the role of epigenetic mechanisms at the interface between the genome and environment, AFB1 and EBV impact the methylome of respectively leukocytes and B cells specifically. Here, we conducted a thorough investigation of common epigenomic changes following EBV or AFB1 exposure in B cells. Genome-wide DNA methylation profiling identified an EBV–AFB1 common signature within the TGFBI locus, which encodes for a putative tumor suppressor often altered in cancer. Subsequent mechanistic analyses confirmed a DNA-methylation-dependent transcriptional silencing of TGFBI involving the recruitment of DNMT1 methyltransferase that is associated with an activation of the NF-κB pathway. Our results reveal a potential common mechanism of B cell transformation shared by the main risk factors of endemic BL (EBV and AFB1), suggesting a key determinant of disease that could allow the development of more efficient targeted therapeutic strategies.

## 1. Introduction

The underlying causes of an active B lymphocyte malignant transformation are attributable to events such as infections or environmental carcinogen exposures that, by interfering with the physiological cell differentiation process within secondary lymphoid organs, predispose the cell to a multistep pathogenic cascade resulting in cancer-related gene deregulation and subsequent lymphoma development [1,2]. Regardless of the nature of the transforming agent, whether an environmental contaminant or a virus, a common oncogenic mechanism consists in the heritable disruption of gene expression patterns in the absence of sequence changes, consistent with the role of epigenetics as an interface between genome and environment [3,4]. Among the wide range of possible B cell hyperproliferative lesions, the differentiation intermediate initiating the malignant transformation defines the lymphoma type; Burkitt lymphoma (BL) is one of the most aggressive and fastest-growing cancers [5,6]. The endemic sub-Saharan African variant (eBL) locally accounts for one in two pediatric cancers and correlates with Epstein–Barr virus (EBV) infection in more than 95% of cases [7,8].

EBV, the first cancer virus ever identified, is a human herpesvirus with a preferential tropism for B lymphocytes, in which it takes advantage in establishing a persistent latent infection [9,10]. Although the infection is predominantly asymptomatic in most of the world’s adult population, EBV is an etiological factor involved in more than 200,000 malignancies per year (lymphomas and carcinomas) and is responsible for 1.8% of all cancer deaths [11]. The hallmark that distinguishes EBV from other human herpesviruses is indeed its ability to provide a proliferative stimulus to initiate the infected cell into the ideal differentiation intermediate for its lifelong viral persistence [12,13]; in this scenario, any other concomitant transforming agent can lead to uncontrolled cell growth [14]. The main transforming EBV factor is the transmembrane protein LMP1, whose structural and functional features mimic a constitutively active CD40 receptor, triggering several signaling pathways crucial for B cell growth and survival and thus EBV-mediated cell transformation [15,16]. Among the downstream targets of LMP1 is the NF-κB pathway, considered the master regulator of innate and adaptive immunity, which generally acts by sensing pathogenic signals and predisposing to appropriate cellular resistance, inducing a proliferative and antiapoptotic program in effector cells [17,18,19]. Beyond the physiological context of B lymphocyte maturation towards an appropriate adaptive immune response, the aberrant EBV-induced NF-κB stimulation drives B cell tumorigenesis [20,21,22]. Profiting from the growth-promoting effects of the latter signaling pathway is achieved in combination with EBV’s appropriate strategies to evade immune surveillance: consistent with latency establishment, a progressive EBV genome silencing reducing the number of viral targets available for immune detection is achieved mainly through extensive viral epigenome alterations [23]. The typical herpesviruses’ biphasic life cycle alternating latent and lytic phases is, in fact, appropriately modulated epigenetically during EBV infection by different gene expression programs, involving the expression of a minimum set of genes, which ensures a balance between episome maintenance and replication requirements and immunogenicity restrictions [24,25]. Thus, later-onset mutations and virus-induced epigenetic changes throughout infection progression potentially may replace the central role of EBV-encoded factors in providing the cell with a selective advantage; the EBV latency is reprogrammed into a complete genome silencing, with the exclusion of the EBNA1 antigen, consistent with its function as episome anchoring protein to the host genome. This occurs in advanced Burkitt lymphoma (latency 1 program), which progressively becomes virus-independent, likely maintained by infection-induced epigenetic changes according to a “hit and run” oncogenesis mechanism, consistent with the absence of expression of key EBV oncoproteins, including LMP1 [26,27]. In this respect, EBV is indeed already known to exploit the host’s epigenetic machinery to modify both its chromatin organization and DNA methylation, and this interplay between viral factors and human epigenetic regulators inevitably affects the host epigenetic landscape as well, further contributing to the oncogenic hijacking of the maturation pathways of the infected B cell [28,29,30]. The relevance of DNA methylation in regulating the EBV life cycle and subsequent cell transformation is well supported by multiple evidence of radically different methylation profiles between healthy and EBV-induced lymphoma or carcinoma samples [30,31]. It is not surprising, then, that the eBL somatic mutation load is lower than its sporadic (EBV-negative) counterpart; however, the eBL specific geolocation also implies the necessary etiological involvement of other concomitant environmental risk factors [32]. In the “lymphoma belt” area, which comprises all the countries across equatorial Africa with the highest incidence of eBL, the combination of climatic conditions of high temperature and humidity that favor fungal proliferation with improper crops harvesting and storage promote heavy food contamination by aflatoxins—mycotoxins produced by Aspergillus fungi [33,34]. Among them, Aflatoxin B1 (AFB1) is considered the most recurrent and harmful and is classified as a class I carcinogen [35,36]. Indeed, studies showed that AFB1 leads to the development of hepatocellular carcinoma as well as growth suppression, immune system modulation, and malnutrition [37,38,39,40]. Although the genotoxicity underlying the transforming potential of aflatoxins has been described as the result of the metabolic conversion occurring predominantly in the liver, therefore justifying the organ specificity of the main associated neoplasm, it has been suggested that AFB1 exposure also contributes to Burkitt lymphoma development; this is partially due to AFB1 effects on impairing immune defenses and favoring EBV infection and cell transformation [33,41]. In addition, AFB1 exposure, which typically starts in utero, impacts B lymphocyte DNA methylation status, potentially predisposing to gene expression reprogramming and subsequent adverse health outcomes. Any significant change in the developing epigenome of the infant is likely to predispose the child to increased disease susceptibility, especially in the presence of a proliferative stimulus provided by EBV infection [42,43].

Since the prognosis of Burkitt lymphoma in equatorial Africa is still poor, mainly due to intensive treatment regimens often applied in resource-poor settings, improving current knowledge of the underlying mechanisms of pathogenesis remains an essential goal in the event that it may suggest new treatment options. In this respect, as it is widely evident that both environmental AFB1 exposure and EBV infection frequently alter DNA methylation status in Burkitt lymphoma, the identification of specific common changes in the methylome could provide valuable biomarkers for predictive and prognostic purposes and suggest more effective therapeutic targets.

In the present study, we, therefore, investigated the global genome methylation status in eBL derived cell lines and human samples seeking a common 5-CpG methylation signature with AFB1 exposure condition. Relevant differentially methylated positions (DMPs) revealed by integrative methylome analysis of the two Burkitt lymphoma predisposing agents were functionally characterized in subsequent mechanistic analyses. Overall, our results provide evidence for the role of TGFBI as a potential cancer-related gene affected by EBV infection and AFB1 exposure-associated hypermethylation.

## 2. Materials and Methods

### 2.1. Cases Selection

A total of 35 BL formalin-fixed and paraffin-embedded (FFPE) samples were used for this study. All cases were retrieved by the Archives of Siena University Hospital and characterized by clinic, morphology, immunophenotype, and cytogenetic consistent with the World Health Organization diagnosis of BL.

Among the 35 BL cases, 20 were EBV positive, whereas 15 were EBV negative. The analysis of the EBV status was performed by in situ hybridization for EBV-encoded RNA (EBER) as previously reported [44]. EBV-positive samples were identified according to the strong nuclear expression of EBV-encoded small RNA genes EBER-1 and -2. A control slide prepared from a paraffin-embedded tissue block containing EBV-positive metastatic nasopharyngeal carcinoma in a lymph node accompanied each hybridization run [45].

### 2.2. Cell Culture and Treatment

Peripheral B cells were purified from blood samples using the RosetteSep human enrichment kit (catalog number 15064; Stem Cell Technologies, Vancouver, BC, Canada). Lymphoblastoid cell lines (LCL) were generated in this study by infection of primary B cells from different donors with EBV. The myeloma-derived RPMI-8226 cells (http://web.expasy.org/cellosaurus/CVCL_0014, accessed on 23 February 2022) and the BL-derived cell lines, including the BL EBV(-) Louckes cell line (http://web.expasy.org/cellosaurus/CVCL_8259, accessed on 23 February 2022), were obtained from the International Agency for Research on Cancer (IARC) Biobank. Primary and immortalized B cells were cultured in RPMI 1640 medium (Gibco, Invitrogen Life Technologies, Cergy-Pontoise, France) supplemented with 10% fetal bovine serum, 100 U/mL penicillin G, 100 mg/mL streptomycin, 2 mM L-glutamine, and 1 mM sodium pyruvate (PAA; Pasching, Austria). EBV (the B95-8 strain) particles produced by culturing HEK293EBVgfp cells were used to infect B cells [46]. The percentage of GFP-positive cells was assessed by fluorescence-activated cell sorting (FACS; FACSCanto system; Becton, Dickinson) and spanned from 10% to 15% at 24 to 48 h postinfection in Louckes and RPMI cells and 60% to 80% when measured at 48 h postinfection in primary B cells. Analysis of the cell cycle and apoptosis (sub-G1 phase) was performed by ethanol fixing of the cells and staining their DNA with propidium iodide at a final concentration of 5 µg/mL. Subsequently, cells were analyzed by FACS. To block DNA methylation, cells were treated with 5-aza- 2=-deoxycytidine (≥97%; catalog number A3656; Sigma-Aldrich, Burlington, MA, USA) at a final concentration of 10 µM for 48 or 96 h. To inhibit the different pathways, cells were treated with the IkBa kinase inhibitor BAY11-7082 (Merck’s Calbiochem, Darmstadt, Germany) at a final concentration of 10 µM (catalog number 420119). Cells were preincubated with the different inhibitors for 2 h. To evaluate the effect of the carcinogen Aflatoxin B1 (AFB1) on DNA methylation, cells were treated with 50 µM AFB1 (catalog number A6636-5MG; Sigma Aldrich, Burlington, MA, USA) for 48 h, negative control of 0.1% DMSO (catalog number D2650 Sigma Aldrich) was used.

### 2.3. qPCR

Total RNA was extracted using an AllPrep DNA/RNA minikit (Qiagen, Hilden, Germany). RNA reverse transcription to cDNA was carried out using RevertAid H Minus Moloney murine leukemia virus reverse transcriptase (Thermo Fisher Scientific, Waltham, MA, USA), according to the manufacturer’s protocol. Quantitative PCR (qPCR) was performed using a MesaGreen qPCR MasterMix Plus for SYBR assay (Eurogentec, Seraing, Belgium). For each primer set, the qPCR was performed in duplicate, and the mRNA levels obtained were normalized to the average mRNA levels of three housekeeping genes (ß-globin, ß-actin, and GAPDH) measured in the same samples. For each PCR, a sample in which the DNA template was replaced with PCR-grade water was included as a negative control. To measure the EBV genome copy number per cell, total DNA was extracted using an AllPrep DNA/RNA minikit (Qiagen) and measured using a NanoDrop spectrophotometer (Thermo scientific, Waltham, MA, USA). Similar amounts of DNA were used as a template for TaqMan PCR, performed according to the protocol described by Accardi et al. [33]. The PCR primer sequences are indicated in Table 1. All the primers used for the first time in the present study were assessed for their efficiency (90% to 110%).

### 2.4. Immunoblotting and Antibodies

Whole-cell lysate extracts were obtained using lysis buffer, as previously described [47]. The cell extracts were then fractionated by sodium dodecyl sulfate-polyacrylamide gel electrophoresis (SDS-PAGE) and processed for immunoblotting using standard techniques. The following antibodies were used for immunoblotting: anti-TGFBI (Beta Ig/TGFBI Antibody; BIO-TECHNE SAS, Minneapolis, MN, USA; NBP1-88606), anti-Tubulin (SIGMA ALDRICH FLUKA SUPELCO; T4026-.2ML), anti-DNMT1 (DNMT1 antibody (60B1220.1) 0.1 mg; NB100-56519 BIO-TECHNE SAS), anti-PIKBa (Phospho IκBα (Ser32/36) (5A5) Mouse mAb; 9246S; Cell Signaling Technology, Danvers, MA, USA), anti-IKBa (Iκbα Antibody; 9242S; Cell Signaling Technology), anti-ß-actin (clone C4; MP Biomedicals, Santa Ana, CA, USA), and anti-GAPDH (Mouse Monoclonal Anti-GAPDH; SC-32233; CLINISCIENCES, Nanterre, France). Images were produced using a ChemiDoc XRS imaging system (Bio-Rad, Hercules, CA, USA).

### 2.5. Immunohistochemistry

Immunohistochemistry analysis for TGFBI was performed on all FFPE 4-µm-thick sections by an automated staining system (Ventana BenchMark Ultra; Roche Diagnostics, Monza, Italy) with anti-TGFBI (Beta Ig/TGFBI Antibody; BIO-TECHNE SAS; NBP1-88606) [28]. An UltraView universal detection kit (Ventana) using a horseradish peroxidase multimer and DAB (as the chromogen) was used. FFPE sections from 10 reactive lymph nodes were used as controls.

### 2.6. Chromatin Immunoprecipitation

ChIP was performed with Diagenode Shearing ChIP and OneDay ChIP kits according to the manufacturer’s protocols. Briefly, 4 × 10^6^ cells were fixed with 1% formaldehyde, lysed, and sonicated 8 min (Bioruptor Pico, Diagenode, Belgium). The following antibodies were used: anti-DNMT1 (catalog number MAB0079; Abnova, Taipei, Taiwan), anti-DNMT3a (AB13888, Abcam), anti-DNMT3b (AB13604, Abcam, Cambridge, UK), and IgG (Diagenode). The eluted DNA was used as a template for qPCR with primers designed on the TGFBI gene. The primers used for quantitative ChIP are listed in Table 1. The value of binding obtained for each antibody was calibrated on the input sample and normalized to the values for IgG.

### 2.7. Bisulfite Modification and DNA Methylation Assessment

Cells were collected and resuspended in a lysis buffer (1% SDS, 0.1 M NaCl, 0.1 M EDTA, 0.05 M Tris pH8 + Proteinase K 500 ug/mL) for 2 h at 55 °C. The DNA saturation and precipitation were made with NaCl (6 M) and isopropanol, then cleaned with 70% ethanol and resuspended in water. DNA was quantified using Quant-IT Picogreen dsDNA reagent (Thermo Fischer Scientific), and 500 ng were bisulfite converted using EZ DNA methylation kit (Zymo) and eluted in 25 µL. Pyrosequencing was performed as described in Hernandez-Vargas, H. et al. [48]. The primers are indicated in Table 2.

The reverse primer was labeled with biotin at the 5′ end.

Methylome profiles were analyzed by the use of HM450 Infinium methylation bead array (Illumina, San Diego, CA, USA), as described in Hernandez-Vargas, H. et al. [42].

### 2.8. DNMTs Assays

Nuclear proteins were extracted by using the Nuclear Extraction Kit (ab1134474 from Abcam) according to the manufacturer’s instructions. Ten µg of nuclear extract was then used to perform the DNMT1 Assay using the DNMT1 Assay Kit (ab113469–Abcam) and the DNMT activity assay (DNMT Activity Quantification Kit (Colorimetric) ab113467-Abcam). DNMT activity was further measured on the ELISA reader Multiskan GO (Thermo scientific) with the software: SkanIt Software Version 5.0.

## 3. Results

### 3.1. EBV and AFB1-Related BL Methylome Profiling Identifies Differentially Methylated Genes

**Figure 1:**
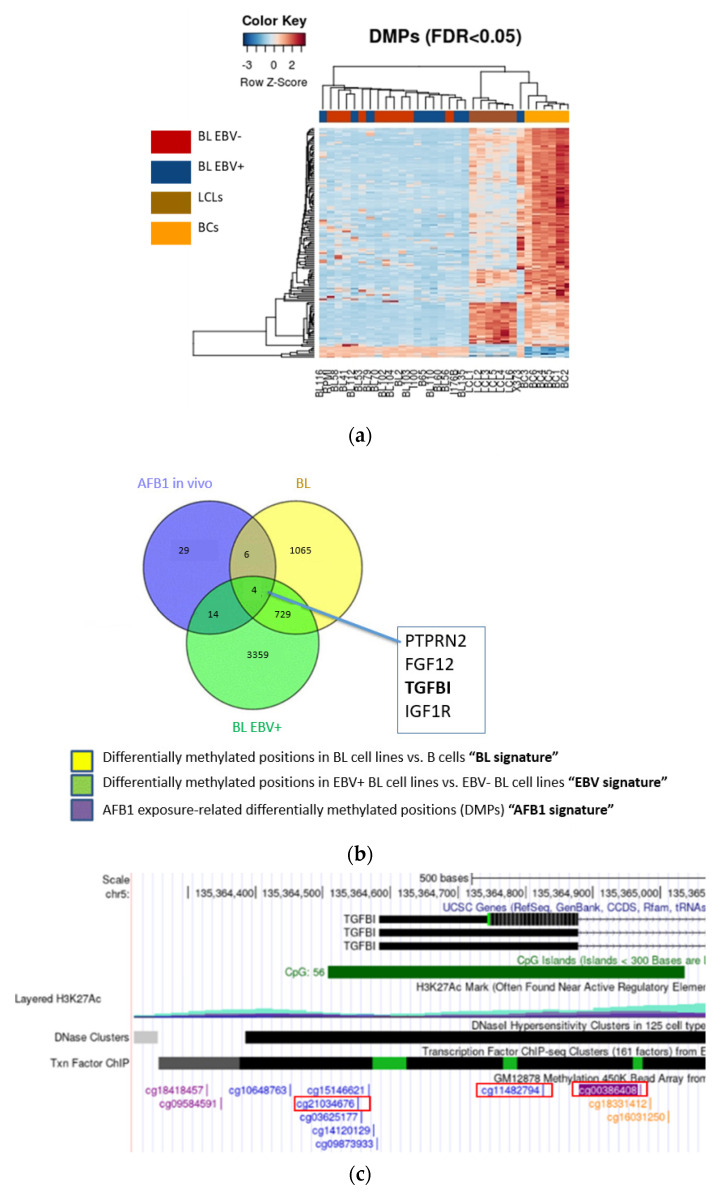
Identifying the methylation signatures of BL, EBV, and AFB1. (**a**) Heatmap of differentially methylated positions (DMPs) in the genome of EBV(+) and EBV(-) BL-derived cell lines, primary B cells, and lymphoblastoid cells (LCL). (**b**) Genes commonly affected by methylation changes identified from the comparative analysis of methylomes associated with the conditions previously illustrated in (**a**) with AFB1 exposure. The Venn Diagram illustrates the intersection of DMPs associated respectively with aflatoxin B1 exposure condition (“AFB1 in vivo”) provided by a publicly available dataset [42], with the eBL-specific ones (“BL EBV+”) identified in a previous study [30] and, ultimately, with the specific signature of B cell transformation state (“BL”). (**c**) TGFBI gene diagram (modified from UCSC Genome Browser) in which the CGs showing differential methylation are highlighted in red boxes.

In order to define an eBL-specific DNA methylation profile, we performed a comparative methylome analysis of primary and in vitro-EBV-immortalised B lymphocytes (LCL cells) with previously identified differentially methylated positions (DMPs) of EBV -positive and negative Burkitt lymphoma-derived cell lines [30]. DNA from the abovementioned samples was interrogated for CpG methylation with the Illumina 450 K bead array (as described in the methods). The resulting heatmap, which illustrates a methylome pattern capable of discriminating samples according to their cell transformation status, highlights DNA methylation changes associated with the EBV-positive lymphoma condition (Figure 1a). Since AFB1 is associated with eBL, we further investigated any common DMP with infection status by integrating publicly available data referring to white blood cell samples from a Gambian population heavily exposed to AFB1 (Figure 1b). Although DNA methylation of peripheral blood leukocytes, and not specifically of B cells, was interrogated in this study, it provided critical evidence of the impact of AFB1 on blood cells methylome [42]. These findings, combined with the epidemiological observations that Burkitt lymphoma is endemic in areas associated with high levels of exposure to aflatoxins, suggest that AFB1 may affect the DNA methylome of B cells and represented a key starting point for our in-depth in vitro investigation. Among the genes mapping to shared EBV-AFB1-associated DMPs, we found Transforming growth factor-ß induced (TGFBI, also known as Beta ig-h3/Big-H3) exhibiting three differentially methylated CpG sites: CpG CG00386408 (referred to as CG00), CpG CG11482794 (CG11), and CpG CG21034676 (CG21) (Figure 1c). Although other genes may also have a valuable role in the pathogenesis of eBL, TGFBI is often deregulated in several cancer types by aberrant DNA methylation mechanisms, and there is currently no evidence regarding its possible role in lymphoma development.

These data reveal that both EBV and AFB1 might influence DNA methylation status on the same CpGs sites within the TGFBI gene.

### 3.2. Validation of the Differential Methylation in the EBV and AFB1-Related BL Cells

**Figure 2:**
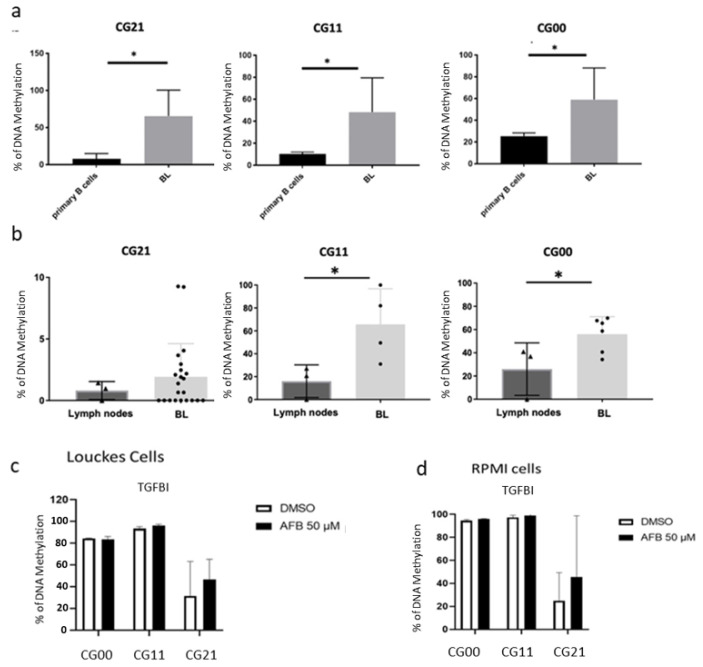
EBV and AFB1-induced hypermethylation of TGFBI promoter. (**a**) Pyrosequencing-based quantification of methylation levels within the TGFBI CpG sites previously identified-CG21, CG11, and CG00–in primary human B cell s and EBV(+) BL derived cell lines (**b**) and in eBL samples (n = 20) vs. healthy patients lymph nodes. Statistical significance was determined by Student’s *t* test (* *p*  <  0.05). Error bars in the graphs represent the standard deviation. (**c**,**d**) Pyrosequencing analysis of the methylation levels within the same CpG sites in EBV(-) BL cells in vitro treated with AFB1 for 48 h; n = 2.

To substantiate the finding of methylome analysis and the AFB1-related in silico data, we aimed to perform validation in vitro. To this end, endemic Burkitt lymphoma-derived cell lines and human eBL samples were subjected to targeted pyrosequencing. The results showed that eBL cell lines exhibited significantly higher methylation levels at the TGFBI CpG sites previously identified as differentially methylated (CG21, CG11, and CG00) in comparison to primary human B cells (Figure 2a). The consistent findings were obtained by evaluating TGFBI methylation levels in human eBL samples and comparing them to those in lymphoid tissue from healthy controls (Figure 2b). Similarly, to validate previous in silico data on AFB1 exposure-associated TGFBI hypermethylation, verifying its specific effect on the B cell subpopulation, EBV-negative cell lines were in vitro-treated with the mycotoxin and analyzed by pyrosequencing at the three CpG sites of interest. The effect of AFB1 exposure on the three CpG sites is slightly evident as it appears in an increased methylation state compared with the untreated control (Figure 2c,d). However, this would not exclude the possibility of a more pronounced effect under chronic in vivo exposure conditions. Taken together, these results validate the findings obtained by the methylome array analysis showing specific changes in DNA methylation levels at three CpG sites within the TGFBI gene in endemic Burkitt lymphoma associated with AFB1 exposure.

### 3.3. The Main EBV Oncoprotein LMP1 Downregulates TGFBI Expression during B Cells Infection

**Figure 3:**
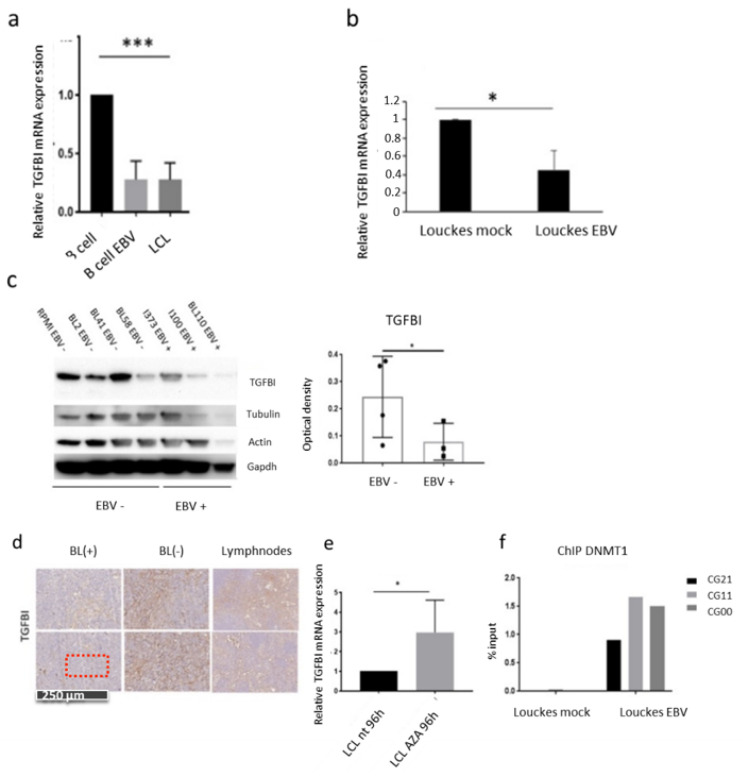

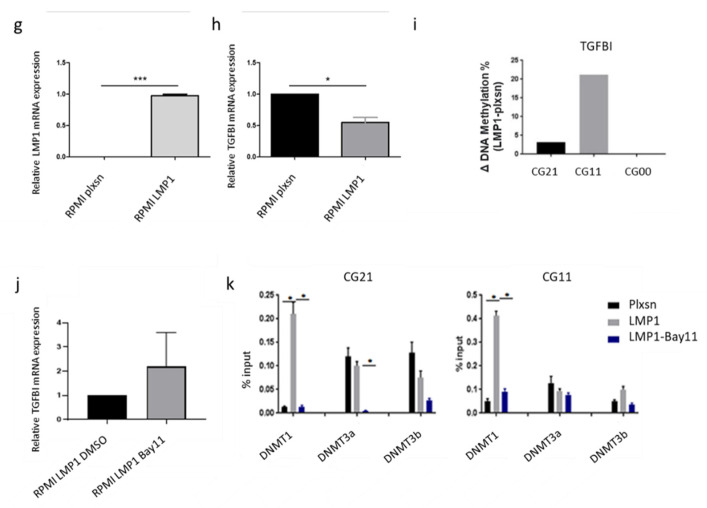
EBV infection leads to TGFBI silencing. (**a**) qPCR analysis of TGFBI mRNA expression levels in primary B cells from 3 independent donors infected with EBV and collected at t = 0 h (B cell), t = 48 h (B cells EBV), and t = 4 weeks (LCL), (* *p*  <  0.05; *** *p* < 0.001) (**b**) qPCR quantification of TGFBI mRNA expression in Louckes cells (EBV (-)) vs. Louckes-EBV. (**c**) Western blot analysis of TGFBI protein expression in several EBV(-) and EBV(+) BL cell lines. The histogram shows the average TGFBI protein levels normalized to the ß-actin, tubulin, and GAPDH signals, measured by Image Lab software (Bio-Rad) in EBV(-) versus EBV(+) BL cells. (**d**) Immunohistochemistry analysis of TGFBI levels in EBV(+), EBV(-) BL, and lymph nodes samples. (**e**) qPCR quantification of TGFBI mRNA in LCL cells treated with the demethylating agent 5-aza-2-deoxycytidine (LCL Aza) or DMSO (n = 3). (**f**) Chromatin Immunoprecipitation (ChIP-qPCR) assessing DNMT1 recruitment on the three CpGs of interest in Louckes vs. Louckes-EBV cells. (**g**,**h**) qPCR analysis of LMP1 and TGFBI expression levels in RPMI cells retrotransduced with an LMP1-encoding vector (pLXSN-LMP1) or pLXSN. n = 3 (**i**) pyrosequencing quantification of TGFBI methylation within the CpGs CG21, CG11, and CG00 in RPMI-LMP1 and RPMI-pLXSN (n = 2). (**j**) qPCR analysis of TGFBI mRNA levels in RPMI-LMP1 cells treated for 2 h with BAY11-7082 (Bay11) (10 µM) (n = 3). (**k**) ChIP-qPCR quantification of DNMT1, DNMT3a, and DNMT3b enzymes recruitment on CG21 and CG11 sites in RPMI-LMP1 or RPMI-pLXSN (n = 3).

To assess whether the EBV-associated increase in DNA methylation at TGFBI sites may impact its gene expression, we monitored its mRNA expression throughout the course of infection. For this, primary B cells isolated from three independent donors were infected in vitro with EBV and then harvested at different time points during the infection until cell immortalization, followed by TGFBI mRNA expression levels analysis (RT-qPCR). We found that upon infection, the level of TGFBI expression is drastically reduced, and the downregulation is maintained in LCL cells originating from the EBV-induced immortalization of B cells (Figure 3a). In order to exclude that the downregulation effect was due to the B lymphocyte activation process itself, resulting from the viral stimulation of cell receptors, we infected with EBV the Louckes cell line and compared the levels of TGFBI expression (RT-qPCR) with the uninfected parental cells (Louckes, BL EBV(-). Here, we found that the presence of the virus alone is clearly sufficient to significantly reduce TGFBI expression (Figure 3b). This downregulation was also seen at the protein level by comparing different available endemic (EBV(+)) or sporadic (EBV(-)) Burkitt lymphoma cell lines. While the TGFBI signal becomes progressively weaker in immunoblot, the respective average from EBV-positive or negative conditions accentuates the significant difference in expression also at the post-transcriptional level. (Figure 3c and Figure S1). These in vitro findings were then reconfirmed on human eBL samples by immunostaining (Figure 3d), showing weak TGFBI staining in 18/20 EBV(+) BL samples compared with BL(−) or healthy lymphoid tissue samples, suggesting the transformation status associated with EBV infection to reduce TGFBI protein expression in vivo.

To further explore the possible involvement of DNA methylation as a putative viral mechanism of TGFBI transcriptional silencing, we treated LCL cells with the demethylating agent 5-azacytidine (Aza) and analyzed TGFBI expression levels by RT-qPCR. As shown in Figure 3e, treatment of LCL cells with DNA methylation inhibitor led to a significantly higher TGFBI expression when compared with their untreated counterparts, consistent with previous reports demonstrating that gene demethylation induced by Aza can effectively reactivate a transcriptionally silent gene [49].

As it is already known that EBV influences DNMT1 activity, we aimed to assess whether an EBV infection results in increased recruitment of the methyltransferase to the TGFBI gene [28,50]. For this, we performed ChIP-qPCR in EBV-infected Louckes cells and in EBV-negative control cells. The results (Figure 3f) showed more recruitment of DNMT1 on the three differentially methylated CpGs of the TGFBI promoter in the EBV-positive cells compared with the EBV negative cells.

Finally, to gain a better insight into the mechanisms of EBV-mediated TGFBI silencing, we assessed the possible involvement of the Latent Membrane Protein (LMP1), the EBV main oncoprotein, which we previously found to influence DNMT1 [28]. We generated an EBV-negative RPMI cell line stably expressing the viral gene LMP1, which allowed us to estimate both TGFBI expression levels and the relative enrichment of DNMT1 recruitment on the three CpG sites in the presence or absence of the viral transformation factor. LMP1 was efficiently expressed in the stably transfected RPMI cells, as shown in Figure 3g. The RT-qPCR analysis revealed a reduction of the expression level of TGFBI in the RPMI-LMP1 cells compared with negative control (cells transduced with the empty retroviral vector pLNSX) (Figure 3h). This suggests the central role of LMP1 in EBV-mediated TGFBI gene silencing. Since LMP1 expression in eBLs is progressively lost and the fact that its functions, including stimulating cell proliferation, may be compensated by mutations or epigenetic changes, it is possible that this LMP1-dependent mechanism may play a role in the early stages of pathogenesis [24,26,27,51]. Moreover, immunoprecipitation experiments (ChIP-qPCR) reconfirmed increased recruitment of DNMT1 on the CpG sites of interest within the TGFBI gene in RPMI-LMP1 cells compared with the pLXSN control (Figure 3i). Overall, these results indicate the EBV mechanism of TGFBI silencing involves the viral protein LMP1, which appears to increase local recruitment of DNMT1 on the three CpG sites of interest.

In addition, we aimed at identifying the signaling pathways involved in this regulation. As the EBV protein LMP1 is known to stimulate the NF-κB pathway, we verified its possible involvement in TGFBI expression regulation in RPMI-LMP1-expressing cells. After the in vitro treatment of the cells with the NF-κB enzymatic inhibitor Bay11, TGFBI expression was measured by RT-qPCR. We found that the treatment with Bay11 resulted in the restoration of TGFBI expression (Figure 3j), providing the support for the involvement of the NF-ΚB signaling pathway in the EBV-mediated regulation of TGFBI. Next, to test whether NF-κB signaling is involved in the recruitment of DNA methyltransferases (DNMT1, DNMT3a, or DNMT3b) to the TGFBI identified DMPs (CG21 and CG11 sites), we performed ChIP-qPCR quantification of DNMT1 local recruitment in RPMI-LMP1 cells treated with the inhibitor Bay11. As shown in Figure 3k, the Bay-mediated inhibition of the NF-κB pathway reduced DNMT1 recruitment without affecting the other DNMTs (DNMT3a and 3b), suggesting that DNMT1 is the methyltransferase responsible for the methylation of the CpG on the TGFBI promoter. In conclusion, TGFBI downregulation appears to be a consequence of EBV infection and, more specifically, of LMP1 expression, which activates the NF-κB pathway and locally recruits the DNA methyltransferase DNMT1 to the CpG sites of interest.

### 3.4. AFB1 Enhances the EBV-Induced TGFBI Downregulation

**Figure 4:**
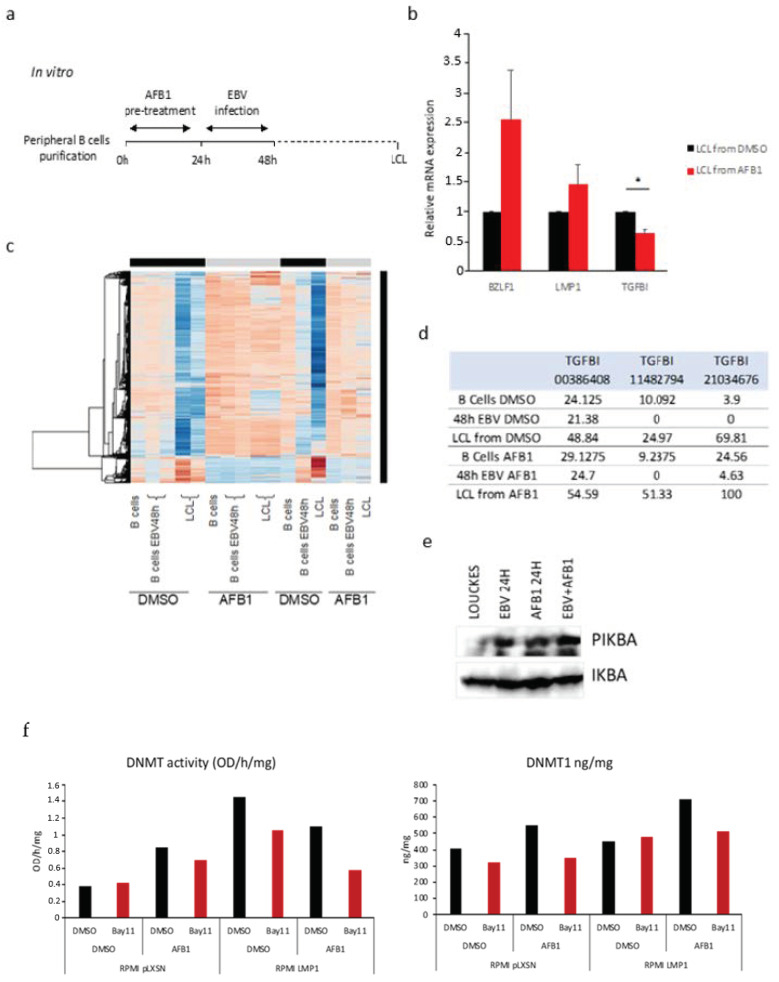
EBV and AFB1 share a common pathway to regulate TGFBI. (**a**) Schematic representation of the in vitro combined AFB1-exposure and EBV-infection-mediated primary B cells immortalization experiment. (**b**) qPCR quantification of TGFBI mRNA expression levels in LCL obtained as explained in (**a**) (* *p*  <  0.05). (**c**) Heatmap of CG methylation levels in B cells at different stages of EBV-mediated immortalization, in presence or absence of concomitant AFB1 in vitro exposure. Independent experiments from different B cells donors. (**d**) pyrosequencing-based analysis of TGFBI methylation levels within the three CpGs of interest at different time points of EBV-induced B cells immortalization with concurrent AFB1/DMSO treatment. (**e**) Immunoblotting quantification of Phospho-IκBα (PIKBA) and IκBα (IKBA) levels in Louckes vs. Louckes-EBV treated or not with AFB1 50 µM. (**f**) The enzymatic assays-based measure of DNMTs activity (left graph (OD/h/mg)) and DNMT1 quantity in the nucleus (right graph (ng/mg)) in RPMI-LMP1 vs. RPMI-pLXSN cell lines treated with AFB1/DMSO and with Bay11/DMSO.

Because our results revealed the differential methylation shared by the two eBL risk factors (EBV and AFB1) (Figure 1), we next investigated the combined effect of EBV infection and AFB1 exposure in deregulating TGFBI. To recapitulate the presumed natural order of exposure to the two risk factors in subequatorial African countries, where AFB1 exposure starts in utero, and EBV infection occurs in early postnatal life, we treated primary B lymphocytes from three different healthy donors with AFB1 in vitro for 24 h prior to EBV infection [52,53]. Cells were subsequently maintained in culture until immortalization (LCL cell lines) (Figure 4a). The derived immortalized LCL cells were then subjected to RT-qPCR to evaluate TGFBI mRNA expression levels as well as the expression of the viral genes LMP1 and BLZF1 (to assess the infection status in the presence of AFB1). Both the levels of LMP1, a latency gene encoding for the major viral oncoprotein, and BZLF1, a key regulator of latent-to-lytic cycle transition and expressed in the prelatency state following de novo infection, increased after AFB1 treatment. This result was expected, as previously reported, that AFB1 enhances EBV infection potential and transforming ability [33] and provided reliable evidence for the observed TGFBI downregulation. Here, the pretreatment with AFB1 led to a significant decrease of TGFBI expression in the resulting lymphoblastoid cells, compared with the unexposed control (DMSO) (Figure 4b).

Furthermore, the methylome analysis of previously generated LCLs showed a distinct pattern in the combined EBV infection-AFB1 exposure condition compared to infection alone one (Figure 4c), suggesting that AFB1 exposure of EBV-infected B cells may corroborate the viral contribution to the host DNA methylome deregulation. To validate these findings, we focused on the CpG sites within the TGFBI gene that were found differentially methylated in genome-wide methylation profiling of LCLs generated in the presence of AFB1 exposure. The targeted pyrosequencing revealed the methylation status to be higher in AFB1-exposed LCLs than in control (Figure 4d), as further evidence of the enhanced impact of the combined presence of the two transforming factors in the context of B cell immortalization.

We then explored the role of NF-κB -mediated recruitment of the DNMT1 in the context of AFB1 exposure. First, to confirm the activation of the NF-κB pathway after AFB1 treatment, we assessed in immunoblot the levels of IKBα phosphorylation in Louckes cells in EBV-infected, AFB1-treated, or simultaneously infected–exposed cells. We found that the PIKBα signal was markedly higher at 24 h post-infection, 24 h post-AFB1 treatment, and in the combined infected–exposed cells compared with Louckes control cells (Figure 4e), indicating that AFB1 treatment, similar to EBV infection, results in activation of NF-κB. Finally, we investigated the influence of both EBV LMP1 and AFB1 in modulating DNMT1 activity and expression by means of enzymatic assays. RPMI-LMP1 and RPMI-pLXSN cells were concurrently treated with AFB1, and Bay11 and the nuclear extracts were screened in ELISA-like assays to estimate DNMT1 activity (expressed in OD/h/mg) and a quantitative measure of the enzyme abundance. The results showed a marked increase of both DNMT1 abundance in the nucleus and an increase of DNMT1 activity in LMP1-expressing and AFB1-exposed conditions, while the Bay11 treatment led to reduced DNMT1 levels compared with the respective DMSO control (Figure 4f). Together, these findings provide evidence of a common molecular mechanism employed by both eBL risk factors in deregulating TGFBI that involves triggering of the NF-κB signaling pathway, which regulates DNMT1 protein amount and catalytic activity.

## 4. Discussion

In the current study, we identified a shared molecular signature of the main endemic Burkitt lymphoma risk factors (EBV and AFB1) that could potentially reveal a key determinant of the disease, thus suggesting valuable biomarkers leading to early diagnosis or the development of more effective treatment strategies.

Consistent with the ability of both EBV and AFB1 to induce relevant changes in the methylome of B lymphocytes, and with DNA methylation itself being a recurrent mechanism of tumor suppressors silencing in cancer, we first performed a methylome analysis of eBL cells and samples vs. primary or immortalized B cells (LCL) to identify an eBL methylation pattern [30,42,54]. In line with human epigenome rewiring being considered as the main EBV transformation mechanism involving the interaction between host epigenetic regulators and virus-encoded proteins, previous studies already highlighted the role of EBV-induced host DNA methylation changes in disrupting the expression of genes with a known or potential role in lymphomagenesis [25,28,30]. However, in the context of Burkitt lymphoma, no one to our knowledge has so far considered a comparative assessment of the key risk factors-associated methylation profiles to explore possible common molecular signatures capable of revealing shared targets and, therefore, putative drivers of BL carcinogenesis. To this end, we performed a comparative analysis of the eBL methylome profile with specific AFB1-associated DMPs [42]. Although the latter were referred to DMPs in the totality of leukocytes and not specifically to B lymphocytes, they provided crucial evidence of the impact of AFB1 on blood cells and represented a key starting point for our subsequent investigation. In addition, epidemiological data supporting the overlap of the endemic spread of Burkitt lymphoma with the regions affected by mycotoxins food contamination further strengthened the hypothesis that AFB1 may affect B cell DNA methylome, potentially relevant to the disease. The comparative analysis provided a set of commonly differentially methylated genes, among which TGFBI (transforming growth factor beta-induced) was selected for our in-depth analysis because the gene is often altered in several cancer, although its involvement in lymphomas has not been described previously [55].

TGFBI was originally identified as a downstream effector of the TGF-β signaling pathway, a key network governing cell proliferation, differentiation, apoptosis, and migration, and playing a key role in tumor suppression in lymphoid cells [56,57,58]. In a broad spectrum of malignancies, a dysregulated TGFBI expression contributes either to cancer initiation or progression, depending on the tumor type and stage: in advanced cancers, TGFBI is often overexpressed, thus acting as an oncoprotein, while during early carcinogenesis, it is usually transcriptionally silenced, consistent with a tumor suppressor function [55]. The recurrent mechanism of gene downregulation in many cancers consists in the TGFBI promoter DNA hypermethylation; given the current lack of information on its possible involvement in the context of BL, we explored the impact of these newly discovered methylation changes within the TGFBI locus on its gene expression [59,60,61,62]. Our study revealed that TGFBI expression is downregulated during EBV infection, in immortalized B cells and in eBL. Moreover, this event appears to be in part a consequence of viral gene expression, thus independent of that cell activation process triggered by the infection, mimicking the physiological antigen-induced B cell differentiation. Although further insights into the function of the dysregulated TGFBI expression in the pathogenesis of eBL are needed, the identified gene silencing mechanism indicated by our study, which is similar to other malignancies having TGFBI role defined as a tumor suppressor, may suggest the same function in the eBL’s carcinogenesis.

Currently, there is only one evidence of TGFBI downregulation induced by a virus, the mutant HBV sW182, and this gene silencing is hypothesized to be essential in the viral-driven hepatocarcinogenesis [63,64]. It is, therefore, possible that TGFBI downregulation through epigenetic mechanism may be a part of common viral strategies to escape from immune surveillance. This is consistent with our finding that LMP1 is the EBV factor responsible for TGFBI hypermethylation and gene downregulation. Many previous studies demonstrated the involvement of LMP1 in modulating the composition of the immunological environment in favor of a long-life infection persistence through regulating the expression of chemokines (CCL17, CCL22), cytokines (IL6, hIL-10), adhesion, and costimulatory molecules (CD18, ICAM, CD80), genes regulating antigen processing and presentation (HLA I, II) [65,66,67,68].

We found that both LMP1 expression and AFB1 exposure trigger the NF-κB pathway, which appears to be a key intermediary in TGFBI deregulation through the recruitment of DNA methyltransferase DNMT1 to TGFBI. This function of NF-κB as an “active repressor” acting through local recruitment of the DNMT1 enzyme and subsequent hypermethylation of tumor suppressor gene promoters under specific cytotoxic stimuli has already been described in several cancer types [69,70,71]. In the specific context of EBV infection, we have already depicted this LMP1–NF-κB–DNMT1 axis as a viral mechanism of deregulation of tumor suppressor genes [28]. However, we cannot exclude the possibility that several other downstream effectors of NF-κB may be activated and therefore have a contribution to TGFBI downregulation such as the polycomb repressor complex 2 subunit EZH2—previously shown to be induced by NF-κB—or that other downstream targets of NF-κB increases DNMT1 expression and activity and therefore recruitment to TGFBI [72]. However, due to a progressive EBV genome silencing occurring throughout infection and subsequent cell transformation, in most eBL cases, LMP1 is not expressed (consistent with a latency I program) [24]. Nevertheless, this event may be functionally compensated both by virus-induced epigenetic changes and mutations arising subsequently during malignant progression; it is, therefore, plausible that LMP1, for example in combination with AFB1, may impair TGFBI expression at the early stages of lymphomagenesis. This supports the “hit and run” theory postulating that the prelatent expression of the viral genes initiates the malignant conversion of the infected B lymphocyte by establishing host genome heritable changes subsequently maintained independently of the continuous presence of the initial triggering stimulus [51].

In the “lymphoma belt” region, the frequent prenatal exposure to AFB1 is estimated to impact the infant’s health by causing immunosuppression (thus, enhancing the susceptibility to subsequent infections) and aberrant DNA methylation [42,43]. At the same time, in these low socio-economic conditions, primary EBV infection occurs within the first years of life (as opposed to late infection during adolescence in high-income countries), and failure of an adequate immune response may allow the virus to promote uncontrolled proliferation of infected B cell, potentially leading to tumor development [53]. In the possible scenario in which an in utero AFB1 exposure with an early-life EBV infection may lead to lymphomagenesis over time, we evaluated the combined effect of both AFB1 and EBV on B cells in vitro. Although AFB1 treatment alone did not produce any significant methylation changes on the TGFBI promoter (Figure 2c,d), the still visible trend of gain in DNA methylation suggests that chronic in vivo exposure may generate DMPs capable themselves to contribute to TGFBI gene silencing. However, given our focus on the eBL pathogenesis, to which EBV infection is an indispensable contributor, our attention has been directed towards considering the two risk factors in combination. By reproducing the natural order of exposure to the two transforming factors, and therefore preceding the in vitro treatment with AFB1 to the de novo EBV infection, we found a marked molecular signature of the co-occurrence of the two risk agents in terms of differential methylome profile of LCLs obtained from primary B cells exposed to the mycotoxin (Figure 4c). This is reflected in increased methylation at the CpG sites, resulting in a stronger TGFBI expression silencing, suggesting that AFB1 exposure synergizes with EBV in affecting TGFBI deregulation to a greater extent. While it is already established that AFB1 impacts EBV infection efficiency and subsequent cell transformation [33], here we show for the first time that, apart from favoring the infection outcomes, AFB1 and EBV share TGFBI as a molecular target. The finding that the two main eBL risk factors compromise TGFBI gene expression through a molecular mechanism of DNA methylation-dependent gene silencing, recurrent in many cancers with a defined tumor suppressor function for TGFBI, suggests for the first time the same possible role in the context of lymphoma.

In addition, consistent with TGFBI function as an extracellular matrix protein involved in mediating cellular interaction, as well as migration, proliferation, apoptosis, and angiogenesis [55], it is plausible that regulation of its secretion levels may play a major role in reshaping the tumor microenvironment in favor of cancer initiation or progression and treatment resistance. Indeed, throughout the process of oncogenesis, the molecular and cell composition of the microenvironment surrounding both the initial expanding cell clone and the progressively more malignant tumor contributes to cancer onset and progression as much as initial transforming events [73,74,75,76,77].

In summary, our study identifies an epigenetic mechanism by which the main eBL risk factors (EBV infection and AFB1 exposure) bring about the silencing of a cancer-related gene (TGFBI) and provides insight into molecular events potentially contributing to eBL development. This molecular insight should open new avenues to investigate a more in-depth assessment of the specific contribution of TGFBI deregulation to eBL pathogenesis as well as their potential as targets for an epigenetics-based strategy for therapy and prevention.

## 5. Conclusions

As endemic Burkitt lymphoma is a highly aggressive pediatric cancer, a thorough investigation of its underlying molecular causes may lead to the identification of new efficient methods of prevention, early diagnosis, and targeted treatment. In our study, we evaluated the potential impact on B cells of the combination of two relevant risk factors, Epstein–Barr virus infection and dietary exposure to Aflatoxin B1. Comparison of changes in infection- and exposure-specific methylomes led to the identification of a shared epigenetic mechanism culminating in the transcriptional silencing of a cancer-related gene, TGFBI. Although further investigation is needed to elucidate the functional role of TGFBI in the pathogenesis of eBL, the silencing mechanism identified in our study is common to a broad spectrum of cancers in which TGFBI acts as a tumor suppressor. Moreover, considering the extracellular localization of TGFBI as a matrix component, its deregulation (already visible at 48 h post-EBV infection and dependent on LMP1 viral oncoprotein expression, which is progressively lost in advanced stages of eBL) might be an early driver of transformation, contributing to a permissive tumor microenvironment for cancer initiation. Further clarification of the biological implications of reduced TGFBI expression may offer improved and more effective treatment regimens in the future.

## Figures and Tables

**Table 1:** Primers used for qPCR and ChIP-qPCR (sequence of primers 5′–3′).

Primer Use and Gene	Forward Primer (5′ to 3′)	Reverse Primer (5′ to 3′)
qPCR		
TGFBI N.1	GTCCACAGCCATTGACCTTT	GAGTTTCCAGGGTCTGTCCA
TGFBI N.2	GCCCTACCACTCTCAAACCT	GTTGACATTGCTGACCAGGG
LMP1	CCAGTCCAGTCACTCATAACG	CCTACATAAGCCTCTCACACT
EBNA1	GGTCGTGGACGTGGAGAAAA	GGTGGAGACCCGGATGATG
BZLF1	AATGCCGGGCCAAGTTTAAGCA	TTGGGCACATCTGCTTCAACAGGA
β2 microglobulin	CTCACGTCATCCAGCAGAGA	CGGCAGGCATACTCATCTTT
ACTIN	CGGCAGGCATACTCATCTTT	TCAACTGGTCTCAAGTCAGTG
GAPDH	GCCAAAAGGGTCATCATC	TGCCAGTGAGCTTCCCGTTC
ChIP-qPCR		
CG11482794	CTCCATGGCCGCTCTCGT	CCCCGACTACCTGACCTTC
CG21034676	AAGGGCTGGGAAAACTGAG	GGCTCCAGGGAAGTGAGAG
CG00386408	CTGCGGAAGGTCAGGTAGTC	AACTCCCTCCCTCTCTCCTT

The TGFBI primers were used in parallel, and the respective results were integrated.

**Table 2:** Primers used for pyrosequencing (sequence of primers 5′-3′).

Pyrosequencing Primers	Sequence			
	Forward Primer (5′ to 3′)	Reverse Primer (5′ to 3′)	Sequencing Primer	Sequence to Analyse
CG11482794	GTTTTGGTTTTGGTTTTGGG	TCCCTCCCTCTCTCCTTCC	GGGTTTYGTTAAG	TYGTTTTATTAG
CG21034676	TGGGTGTTTAGGGTAGTTAGGG	CCCAAAACCAAAACCAAAAC	TAGGGTAGTTAGG	GGYGTAYGGGT
CG00386408	GTTTTGGTTTTGGTTTTGGG	TCCCTCCCTCTCTCCTTCC	TGAATTGGGTTGGG	GGYGTAGGGGA

## Data Availability

All sequencing data supporting the results of this study are accessible at the NCBI Gene Expression Omnibus under accession numbers GSE59592 and GSE92378.

## Supplementary Materials

The following supporting information can be downloaded at: https://www.mdpi.com/article/10.3390/cancers14051284/s1, Figure S1: Original Western Blot figure for Figure 3c.
