# Supplementary material for: Epigenetic Alteration of the Cancer-Related Gene TGFBI in B Cells Infected with Epstein–Barr Virus and Exposed to Aflatoxin B1: Potential Role in Burkitt Lymphoma Development
Source: Cancers (Basel). 2022 Mar 2;14(5):1284. doi: 10.3390/cancers14051284 (PMC8909323; doi:10.3390/cancers14051284)
Supplement: Supplementary file 1 [file cancers-14-01284-s001.zip › cancers-1605225-supplementary.pdf]

Article

Epigenetic Alteration of the Cancer-Related Gene TGFBI in B cells Infected with Epstein–Barr Virus and Exposed to Aflatoxin B1: Potential Role in Burkitt Lymphoma Development

Francesca Manara, Antonin Jay, Grace Akinyi Odongo, Fabrice Mure, Mohamed Ali Maroui, Audrey Diederichs, Cecilia Sirand, Cyrille Cuenin, Massimo Granai, Lucia Mundo, Hector Hernandez-Vargas, Stefano Lazzi, Rita Khoueiry, Henri Gruffat, Zdenko Herceg and Rosita Accardi

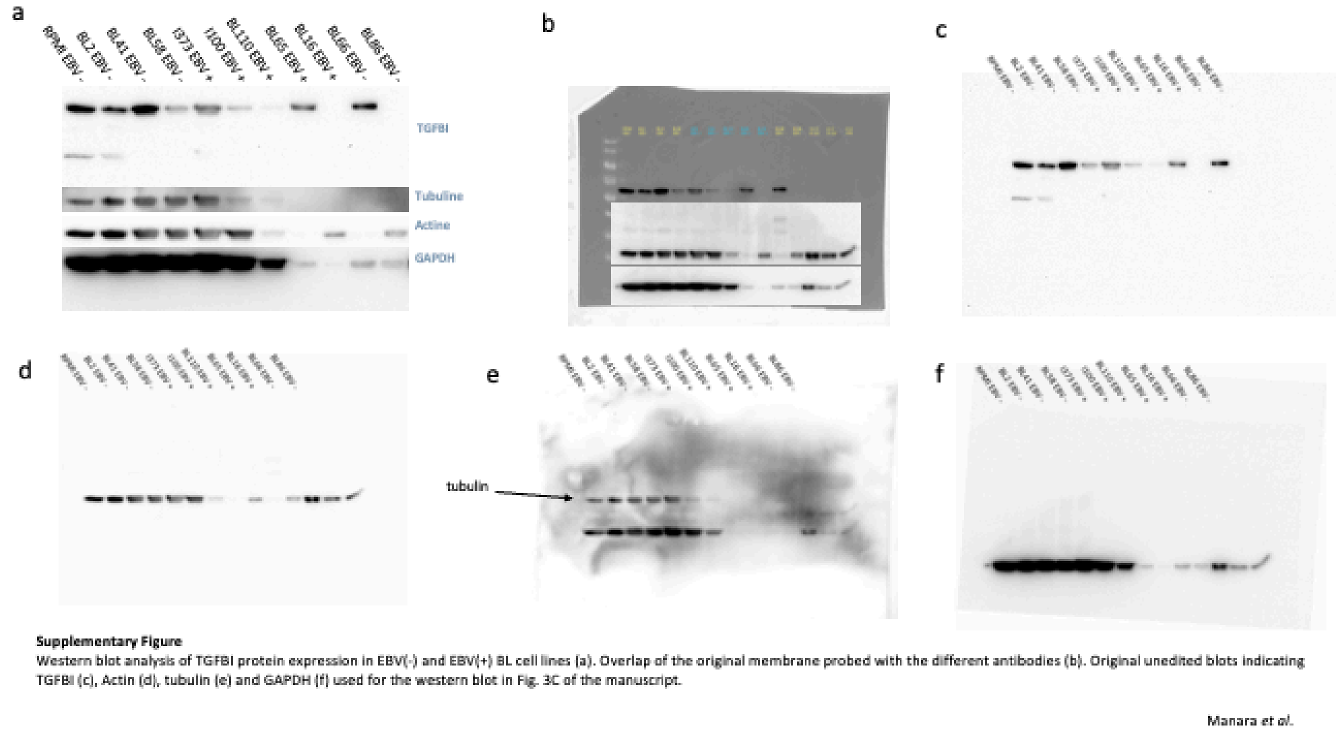

Figure S1. Original Western Blot figure for Figure 3c.
